# The efficacy and safety of modified ultraearly oral hydration for alleviating thirst in patients after thoracoscopic surgery: a prospective randomized controlled trial

**DOI:** 10.1186/s12871-024-02497-7

**Published:** 2024-03-19

**Authors:** Yan Xue, Qian Wang, Hongyu Zhao, Ren Pan, Yanfei Xia, Hongmei Wang, Xiarong Qin

**Affiliations:** 1 Department of Anesthesiology, Zhejiang Hospital, Hangzhou, 310013 Zhejiang Province China; 2 Department of Pain Management, Dongguan Songshan LakeCentral Hospital, Dongguan, 523320 Guangdong Province China

**Keywords:** Postoperative ultraearly oral hydration, Middle-aged and elderly patients, Thoracoscopic surgery, Postanesthesia Care Unit

## Abstract

**Objective:**

Postoperative fasting following thoracoscopic surgery can cause intense thirst and oral discomfort. However, there is currently no research on ultraearly oral hydration (UEOH) in middle-aged or elderly patients after thoracoscopic surgery. The aim of this study was to investigate the effectiveness and safety of UEOH for improving oral discomfort after thoracoscopic surgery.

**Methods:**

This single-center prospective double-blind randomized controlled trial was conducted from April 2022 to November 2023. A total of 64 middle-aged and elderly patients who underwent the first thoracoscopic surgery on the day were enrolled at our institution. Postoperatively, in the Postanesthesia Care Unit (PACU), patients were randomly assigned at a 1:1 ratio to either the UEOH group or the standard care (SC) group. The primary outcome was the patient’s thirst score at 6 h after surgery. Secondary outcomes included the incidence of postoperative oral discomfort; pain scores; the occurrence of adverse reactions such as nausea, vomiting, regurgitation and aspiration; anxiety scores on the first postoperative day; the time to first flatus; and recovery satisfaction scores.

**Results:**

The demographic and surgical characteristics were similar between the two groups. Patients in the UEOH group had lower thirst scores 6 h after surgery than did those in the SC group(16.1 ± 6.70 vs. 78.4 ± 8.42, *P* < 0.01). The incidence of postoperative oral discomfort (*P* < 0.01), anxiety scores on the first postoperative day (*P*<0.05), and time to first flatus (*P*<0.05) were better in the UEOH group. Additionally, the incidences of adverse reactions, such as postoperative nausea, vomiting, regurgitation and aspiration, were similar between the two groups (*P*>0.05).

**Conclusion:**

For middle-aged and elderly patients undergoing thoracoscopic surgery, the use of a modified UEOH protocol postoperatively can improve thirst and promote gastrointestinal recovery without increasing complications.

**Trial registration:**

This single-center, prospective, RCT has completed the registration of the Chinese Clinical Trial Center at 07/12/2023 with the registration number ChiCTR2300078425.

## Background

Thoracoscopic surgery, which involves a minimally invasive approach, wide visual field, minimal bleeding, low complication rate, and rapid postoperative recovery, has become the preferred treatment option in thoracic surgery [1, 2]. During anesthesia for thoracoscopic surgery, double-lumen endotracheal tubes are commonly used. These tubes have a larger diameter and are positioned deeper than standard endotracheal tubes are, causing greater irritation to the vocal cords and pharyngeal region during intubation and extubation. These adverse effects result in increased postoperative dry mouth and thirst, leading to oral discomfort and decreased postoperative comfort. Currently, the conventional approach for addressing postextubation thirst involves moistening the lips with a water-dipped cotton swab, which does not relieve internal oral discomfort. There is insufficient evidence regarding when to resume drinking after surgery. With the introduction of the enhanced recovery after surgery (ERAS) concept, although studies have reported on the efficacy and safety of early postoperative hydration for several surgeries [4–6], the impact and safety of ultraearly oral hydration (UEOH) in alleviating oral and pharyngeal discomfort in thoracoscopic surgery patients, who often experience more severe discomfort and pain than general anesthesia patients and require continuous opioid analgesics, have not been validated. The aim of this article was to investigate the ability of UEOH to relieve oral and pharyngeal discomfort in middle-aged and elderly patients after thoracoscopic surgery and to propose an improved hydration scheme.

## Materials and methods

### Patients

This was a single-center, prospective, double-blind, randomized controlled study. The study received approval from the Ethics Committee of Zhejiang Hospital (Approval No. 47 K of 2022) and was registered with the China Clinical Trial Registry (ChiCTR2300078425). Informed consent was obtained from the patients or their family members before surgery.

The study adhered to the Consolidated Standards of Reporting Trials (CONSORT) guidelines [7]. A total of 64 patients who underwent the thoracoscopic surgery at Zhejiang Hospital from April 2022 to November 2023 were selected for the study. All patients were admitted to the Postanesthesia Care Unit (PACU) postoperatively. The inclusion criterion were patients aged 45 ∼ 80 years, classified as I to III according to the American Society of Anesthesiologists (ASA) classification, who underwent the first thoracoscopic surgery on the day. The exclusion criteria included patients with language and communication barriers, mental illness, preoperative dysphagia, intestinal obstruction, diabetes, a history of postoperative nausea and vomiting, and who refused to participate in the study.

### Study protocol

Anesthesia introduction was performed by administration of 2 mg midazolam, 0.6 ug/kg sufentanil, 0.3 mg/kg Etomidate and 0.7 mg/kg rocuronium bromide. After intrabronchial intubation, mechanical ventilation was supported at an end-tidal CO2 (ETCO2) of 30 ∼ 40 mmHg and SpO2 of 95 ∼ 100% with an oxygen concentration of 50 ∼ 100%. Anesthesia maintenance was performed by administering propofol at 4 ∼ 10 mg/kg/h, titrated to the bispectral index within 40 ∼ 60, remifentanil at 0.1 ∼ 0.4 mg/kg/h, and cisatracurium at 0.06 ∼ 0.12 mg/kg/h. 5–10 ug of sufentanil was administered intravenously before the start of the surgery and 30 min before the end of the surgery, respectively. Trotaxetron was given intraoperatively to prevent PONV before the surgery end.

After anesthesia, patients were positioned in the lateral decubitus position with the operative side up. Using a 1.6 to 6.0 MHz convex array probe, the probe was placed in the sagittal position and scanned laterally from the median line to identify the spinous processes, transverse processes and paravertebral spaces at the T4 and T7 levels. Using an in-plane approach, an 18G puncture needle was inserted into the paravertebral space and 40 ml of 0.25% ropivacaine was injected.

All patients were admitted to PACU after surgery and then randomly divided into a UEOH group and a standard care (SC) group at a 1:1 ratio using a random number table. Patients in both groups were administered thoracic paravertebral nerve blocks combined with PCA postoperative analgesia. Continuous infusion of sufentanil was used for patient-controlled analgesia (PCA) at the end of surgery. Sufentanil 100ug plus tolansetron 10 mg diluted to 100 ml. Parameters of self-controlled analgesia were a continuous dose of 2mL with a lock time of 15 min and a single additional dose of 1 ml. Routine monitoring of arterial blood pressure, heart rate, SpO2, and electrocardiogram was conducted, and a negative pressure aspirator was prepared after admission to the PACU. In the UEOH group, after the removal of the endotracheal tube and recovery of consciousness, the patients’ level of alertness, airway patency, and limb mobility were assessed using the Steward Recovery Scale. When the score reached ≥ 5, trained medical staff used the Thirst Scale Score [8] for evaluation and implemented the hydration protocol. Based on the studies by Wu and others [4, 9], a modified ultraearly hydration protocol was adopted: the head of the bed was elevated 15 ∼ 30°, the patient’s head was turned to one side, and warm sterile water was slowly injected into the mouth in increments using a 30 ml sterile syringe. After the initial 2 ml injection, the swallowing reflex and coughing were closely monitored. If no significant reaction was observed, 5 ∼ 10 ml was administered in increments. After the first hydration, water was given every 2 h, with each session’s total volume not exceeding 50 ml and the smallest volume based on the patient’s willingness. In the SC group, after removal of the endotracheal tube following assessment, the lips were moistened with a cotton swab dipped in warm sterile water, and this process was repeated every 2 h. Six hours after surgery, both groups were allowed to drink water normally.

### Randomization and blinding

The randomization sequence was generated using a random number scale by individuals not involved in the trial. After the allocation list was created, it was placed in an envelope. Upon a patient’s entry into the PACU, the nurses in the resuscitation room opened the envelope to group the patient and administer the corresponding care. All patients received the same amount of water in the same disposable cups and were unaware of their group. Relevant study data were collected by specialised data collectors who were also blinded to the study subgroups.

### Measurements

The primary outcome was the level of thirst at 6 h postsurgery, assessed using the Thirst Scale score, with scores ranging from 0 to 100. The thirst scale score was referenced to the Numerical Rating Scale [10] (NRS) pain score by asking the patient to mark the level of thirst on the NRS scale, with 0 representing no thirst at all and a score of 10 representing extreme thirst, and then multiplying the score by 10 to obtain a thirst score ranging from 0 to 100. Higher scores indicate more severe thirst. Secondary outcomes included the incidence of postoperative oral discomfort; pain scores; the occurrence of adverse reactions such as nausea, vomiting, regurgitation and aspiration; anxiety scores on the first postoperative day; the time to first flatus; and recovery satisfaction scores. Oral and pharengeal discomfort was defined as the presence of one or more symptoms, such as sore throat, dry throat, or hoarseness. Pain was assessed using the NRS, with scores ranging from 0 ∼ 10; higher scores indicate more severe pain. Anxiety was measured using a self-rating anxiety scale [11] with 20 items, where a standard score of < 50 was considered normal, 50 ∼ 59 indicated mild anxiety, 60 ∼ 69 indicated moderate anxiety, and ≥ 70 indicated severe anxiety. Recovery satisfaction was scored from 0 ∼ 100, with higher scores indicating greater satisfaction. The incidence of nausea and vomiting was defined as the occurrence of one or both conditions.

### Statistical analysis

Based on previous studies, the standard deviation for the thirst score was set at 38.2, with an anticipated mean difference of 33.9 between the two groups [12]. For this study, a type I error rate of 0.05 (two-sided), a power of 90%, and an estimated dropout rate of 10% were assumed. Consequently, a total sample size of 64 patients was necessary for this study [13].

The statistical analysis was performed using SPSS software version 22.0. The Shapiro‒Wilk test was used to assess the normality of continuous variables. For normally distributed quantitative data, the mean ± standard deviation (x ± s) was used for representation, and comparisons between groups were conducted using the independent samples t test. Nonnormally distributed quantitative data are presented as medians (interquartile ranges), and the Mann‒Whitney U test was applied for group comparisons. Categorical data are expressed as frequencies and percentages (%), and the chi-square (*χ2*) test was used for comparisons between groups. A *p* value of less than 0.05 was considered to indicate statistical significance.

## Results

**Fig. 1:**
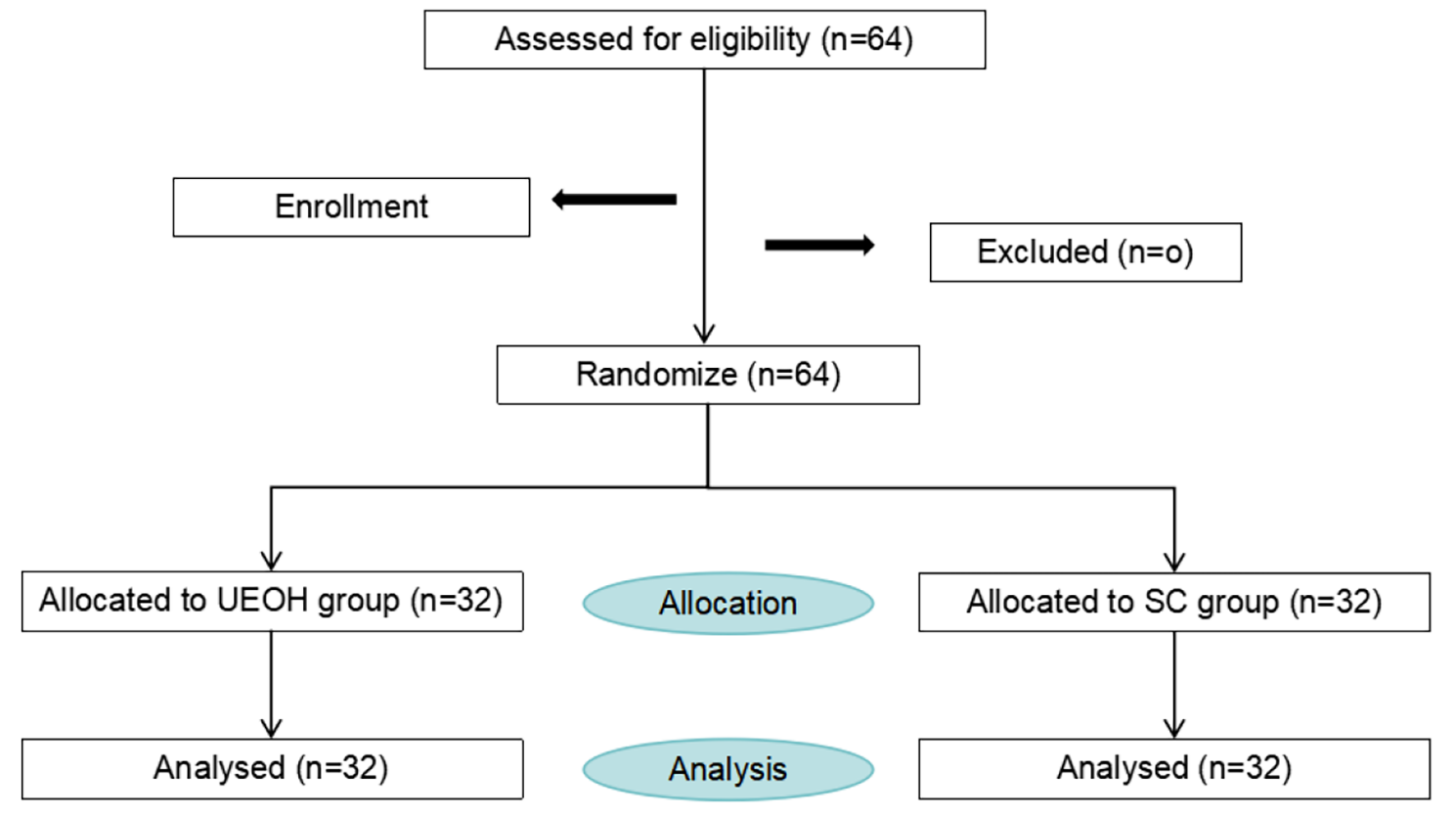
Study flowchart

A total of 64 patients were included in this study, with no dropouts. There were 32 patients each in the UEOH group and the SC group. All patients in the UEOH group tolerate drinking water. Statistical analysis was also conducted on the data collected from these patients (Fig. 1). There were no statistically significant differences between the two groups in terms of patient age, gender, weight, anesthesia duration, intraoperative fluid volume, ASA classification, intraoperative opioid usage, postoperative MAP, surgery duration, intraoperative blood loss or surgery type (Table 1).

**Table 1:** Patients characteristics between the two groups

Items	UEOH group(*n* = 32)	SC group (*n* = 32)	*P*-value
**Age** (year), mean ± SD	66.7 ± 5.64	65.1 ± 5.90	0.256
**Gender** (n, %)			0.211
Male	19 (59.4)	14 (43.8)	
Female	13 (40.6)	18 (56.2)	
**Weight** (kg), mean ± SD	64.3 ± 9.04	61.6 ± 7.29	0.222
**Anesthesia duration** (min), mean ± SD	184.5 ± 79.57	178.3 ± 64.05	0.734
**Intraoperative infused fluids volume** (ml)	900.0 (812.5 ∼ 1262.5)	900.0 (850.0 ∼ 1000.0)	0.973
**ASA classification** (n,%)			0.356
I	0(0)	1(0.03)	
II	32(100)	30(0.94)	
III	0(0)	1(0.03)	
**Thirst Score of post extubation** (mean ± SD)	66.0 ± 15.76	62.7 ± 14.76	0.389
**The incidence of oral discomfort of post extubation** (n, %)	26(81.3)	25(78.1)	0.756
**Anxiety scores before surgery** (mean ± SD)	64.6 ± 9.39	60.5 ± 8.83	0.770
**Sufentanil** (ug)	45.0(40.0 ∼ 50.0)	42.5(35.0 ∼ 50.0)	0.442
**Remifentanil** (mg)	1.0(0.65 ∼ 1.53)	1.0(0.73 ∼ 1.40)	0.929
**Postoperative MAP** (mmHg), mean ± SD	87.9 ± 9.65	85.9 ± 9.56	0.267
**Surgery duration** (min)	122.0(75.5 ∼ 185.5)	125.0(80.3 ∼ 177.0)	0.979
**Intraoperative blood loss** (ml)	25.0(10.0 ∼ 100.0)	30.0(10.0 ∼ 100.0)	0.870
**Surgery type** (n, %)			0.661
Radical resection	2(6.3)	2(6.3)	
Segmentectomy	2(6.3)	5(15.6)	
Wedge resection	12(37.5)	10(31.3)	
Lobectomy	13(40.6)	10(31.3)	
Thymomectomy	3(9.4)	5(15.6)	

**Fig. 2:**
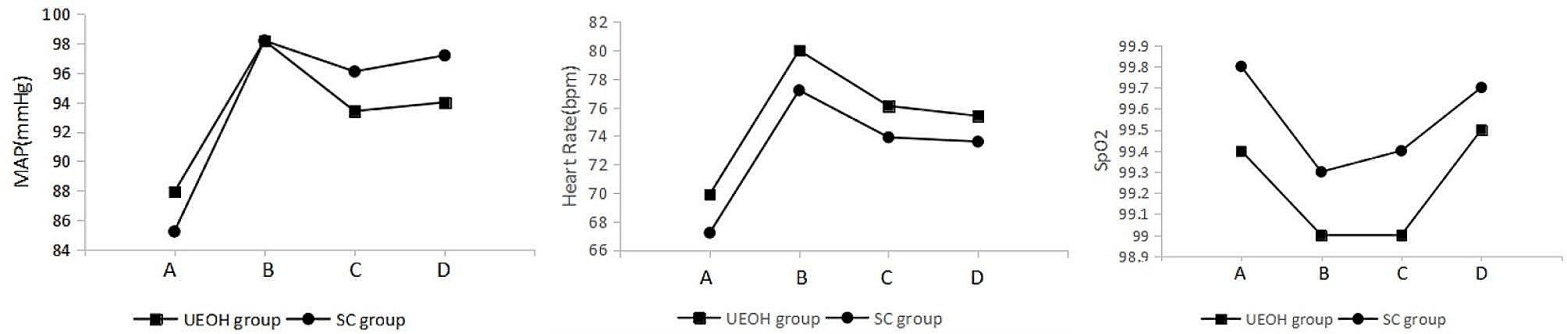
Changes in MAP, Heart Rate and SpO2 during the PACU stay (A - PACU admission, B - at extubation, C − 10 min after extubation, D - discharge from PACU )

Blood pressure, heart rate and SpO2 were monitored in the PACU and compared at 4 time points: at admission to the PACU, at extubation, 10 min after extubation and at discharge from the PACU. The changes in mean arterial pressure (MAP), heart rate and SpO2 were comparable between the two groups, with no significant differences (*P* > 0.05) (Fig. 2).

At 6 h postsurgery, the UEOH group had significantly lower thirst scores than did the SC group (16.1 ± 6.70 vs. 78.4 ± 8.42, *P* < 0.01). The UEOH group also had better outcomes in terms of thirst scores and the incidence of oral discomfort at the time of discharge from the PACU, the incidence of oral discomfort at 6 h postsurgery, anxiety scores on the first postoperative day, and time to first flatus than did the SC group (*P* < 0.05). There were no significant differences between the two groups in pain scores at 6 h postsurgery or recovery satisfaction scores (*P*>0.05) (Table 2).

**Table 2:** Comparison of postoperative comfort between the two groups

Items	UEOH group(*n* = 32)	SC group (*n* = 32)	*P*-value
**Primary outcome**			
**Thirst Score** (mean ± SD)			
6 h after surgery	16.1 ± 6.70	78.4 ± 8.42	<0.01
**Secondary outcome**			
**Thirst Score** (mean ± SD)			
Discharge from the PACU	25.0 ± 11.41	73.4 ± 12.31	<0.01
**The incidence of oral discomfort** (n, %)			
Discharge from the PACU	8(25.0)	27(84.4)	<0.01
6 h after surgery	4(12.5)	28(87.5)	<0.01
**Pain scores** (mean ± SD)			
6 h after surgery	2.50(2.00 ∼ 3.45)	3.00(2.00 ∼ 4.00)	0.603
**Anxiety scores** (mean ± SD)			
Postoperative day 1	50.0 ± 8.05	56.7 ± 1059	0.015
**Theresuscitation satisfaction scores** (mean ± SD)	90.6 ± 5.76	88.4 ± 6.81	0.158
**Time of first postoperative expiration** (mean ± SD)	17.0 ± 3.98	18.9 ± 2.64	0.030

The incidence of nausea and vomiting in the UEOH group was not greater than that in the SC group (*P*>0.05). Neither group experienced adverse reactions such as regurgitation or aspiration (Table 3).

**Table 3:** Comparison of adverse reactions between the two groups

Items	UEOH group(*n* = 32)	SC group (*n* = 32)	P-value
**The incidence of nausea and vomiting** (n, %)			
Discharge from the PACU	3(9.4)	3(9.4)	1.000
6 h after surgery	4(12.5)	5(15.6)	0.719
**The incidence of regurgitation and aspiration** (n, %)	0	0	NA

NA: not applicable

## Discussion

In this study, we found that modified UEOH after thoracoscopic surgery in middle-aged and elderly patients can relieve thirst at 6 h postsurgery, reduce the incidence of oral discomfort, alleviate postoperative anxiety, and promote gastrointestinal motility without increasing the incidence of adverse reactions such as nausea, vomiting, regurgitation or aspiration.

Postoperative thirst and dry mouth are among the primary stressors that decrease comfort, yet these symptoms are often overlooked. During recovery from general anesthesia, as patients regain consciousness, in addition to pain relief, the demand for drinking water becomes a primary concern. In a previous study by Wu et al. [5] 86.8% of patients had a desire for water after surgery. The European Society of Anesthesiology’s guidelines for preoperative fasting in children and adults also recommend immediate hydration postelective surgery according to patient desire [14]. In this study, drinking small amounts of water in divided doses beginning after extubation in the PACU was effective in relieving patients’ thirst and oral discomfort for 6 h after surgery. Yin and Wu et al. [4, 8] also concluded in their adult and pediatric studies that in non-gastrointestinal surgery, patients’ gastric motility returns to baseline levels in a short period of time, which allowing early restoration of hydration. It is not only safe and well tolerated, but also significantly less thirsty than delayed oral hydration. In addition to relieving thirst, early oral hydration acts as a mouth cleanser. It reduces oral discomfort by flushing out oral secretions and reducing oral bacterial residue, eliminating oral odor, and reducing sore throat [15]. Our study demonstrated that UEOH can alleviate thirst, this may be because early, moderate water intake stimulates saliva production, maintaining oral moisture. This approach is particularly beneficial for elderly people whose organ functions and glandular secretions decline, increasing the effectiveness of early oral hydration in relieving thirst and enhancing postoperative comfort. Higgins et al. [16] suggest that medical staff should consider means of improving prioritisation and cohesive delivery of person-centred hydration care.

The traditional concept of postoperative fasting assumes that, due to the residual effects of anesthetics and the inhibitory effects of pain on the gastrointestinal tract, particularly in elderly patients, early resumption of diet can lead to adverse reactions such as nausea, vomiting, regurgitation and aspiration [3, 17]. To ensure patient safety, nongastrointestinal surgery patients must typically fast for at least 6 h postoperatively, leaving some patients to endure significant thirst and oral discomfort during this time. Wu et al. [4] conducted a single oral fluid intake procedure early postoperatively and did not resume hydration within 6 h, assessing only the 20-minute posthydration thirst relief rate; thus, the relief of thirst within 6 h postoperatively was unclear. Çalişkan et al. [9] studied a single early postoperative hydration volume of 200 ml. Considering the risk of consuming 150–200 ml of warm water immediately, our study adopted a modified hydration scheme to alleviate thirst and reduce the risk associated with hydration within 6 h after surgery. The total hydration volume was divided into three sessions, with no more than 50 ml per session, during which the patients were observed within 6 h.

The ERAS concept suggests that early postoperative hydration in small amounts can reduce the duration of intestinal paralysis and promote recovery of gastrointestinal function, enabling the gastrointestinal tract to return to its preoperative level more rapidly [9, 18]. Our study showed that in the UEOH group, standardized hydration occurred after extubation in the PACU. During the PACU stay and within 6 h postoperatively, there was no significant difference in the incidence of nausea or vomiting between the two groups, and no complications, such as regurgitation or aspiration, occurred. The results of several other studies in cardiothoracic surgery, cesarean section and non-gastrointestinal surgery have also confirmed that there is no significant difference in the incidence of nausea and vomiting when early oral hydration is given after surgery [3, 19, 20].

Additionally, the time to first flatus was shorter in the UEOH group than in the SC group (*P* < 0.05), indicating that early postoperative hydration not only does not increase related complications but can also promote early recovery of gastrointestinal function; this may be because, on the one hand, drinking water stimulates the central nervous system, which excites digestive glands via the autonomic nervous system, promoting digestive fluid secretion and intestinal motility. On the other hand, acetylcholine released by autonomic nerve endings excites gastrointestinal smooth muscles, promoting gastrointestinal motility and accelerating functional recovery. This mechanism lays the foundation for further early postoperative nutritional intake, preventing nutritional deficits, and accelerating early recovery.

Although thoracoscopic surgery is minimally invasive, the incisions in this surgery are close to the intercostal nerves, and postoperative placement of drainage tubes can cause acute pain, intensifying stress responses such as restlessness and anxiety. Prolonged postoperative fasting lowers comfort, exacerbates pain, and exacerbates adverse experiences such as anxiety, hindering early recovery. Pimenta et al. [21] suggested that prolonged postoperative fasting increases patient discomfort and the risk of metabolic reactions, infections, and insulin resistance. Robertson et al. [22] demonstrated that early postoperative hydration could shorten hospital stays, alleviate economic pressure, and somewhat reduce patient anxiety. Our study also confirmed that UEOH can improve postoperative anxiety symptoms, possibly through effective intraoperative and postoperative analgesia, resulting in no significant difference in postoperative pain between the two groups. The ERAS concept advocates early oral feeding as the preferred nutritional method for every patient postoperatively. UEOH can mitigate pain and reduce negative experiences such as restlessness and anxiety, enhancing postoperative comfort and satisfaction.

No difference in anesthesia recovery satisfaction was observed between the two groups; this may be because both groups had high satisfaction scores upon PACU discharge, as postoperative hydration is only one aspect of recovery room care, and the overall quality of care in the recovery room was recognized by patients.

However, It is necessary to include more patients to improve statistical significance and confirm the results obtained.

A limitation of this study is that oral rehydration and moistening the lips with a cotton swab dipped in water are two different methods, and this may not have been done in a strictly blinded manner.

## Conclusions

In conclusion, providing modified UEOH to middle-aged and elderly patients after thoracoscopic surgery in the PACU can significantly alleviate thirst and oral discomfort, reduce postoperative anxiety and other adverse experiences, enhance postoperative comfort, and promote early gastrointestinal recovery without increasing adverse reactions. Modified UEOH has a positive effect on rapid postoperative recovery. This study demonstrated the benefits and safety of UEOH for patients, but additional research is needed to confirm these findings.

## Data Availability

The datasets used and/or analysed during the current study are available from the corresponding author on reasonable request.

## Abbreviations

UEOH: Ultraearly oral hydration
SC: Standard care
PACU: Postanesthesia Care Unit
NA: not applicable
MAP: mean arterial pressure
ERAS: enhanced recovery after surgery
ASA: American Society of Anesthesiology
ChiCTR: Chinese Clinical Trial Registry
NRS: Numerical Rating Scale
